# Dietary iron intake, body iron stores, and the risk of type 2 diabetes: a systematic review and meta-analysis

**DOI:** 10.1186/1741-7015-10-119

**Published:** 2012-10-10

**Authors:** Wei Bao, Ying Rong, Shuang Rong, Liegang Liu

**Affiliations:** 1 Department of Nutrition and Food Hygiene, Hubei Key Laboratory of Food Nutrition and Safety, School of Public Health, Tongji Medical College, Huazhong University of Science and Technology, 13 Hangkong Road, Wuhan 430030, P.R. China; 2 Ministry of Education Key Laboratory of Environment and Health, School of Public Health, Tongji Medical College, Huazhong University of Science and Technology, 13 Hangkong Road, Wuhan 430030, P.R. China

## Abstract

**Background:**

Excess iron has been shown to induce diabetes in animal models. However, the results from human epidemiologic studies linking body iron stores and iron intake to the risk of type 2 diabetes mellitus (T2DM) are conflicting. In this study, we aimed to systematically evaluate the available evidence for associations between iron intake, body iron stores, and the risk of T2DM.

**Methods:**

A systematic search of the PubMed/MEDLINE and EMBASE databases to the end of 22 April 2012 was performed, and reference lists of retrieved articles were screened. Two reviewers independently evaluated the eligibility of inclusion and extracted the data. Pooled relative risks (RRs) and 95% confidence intervals (CIs) were calculated using random-effects models.

**Results:**

We reviewed 449 potentially relevant articles, and 11 prospective studies were included in the analysis. A meta-analysis of five studies gave a pooled RR for T2DM of 1.33 (95% CI 1.19 to 1.48; *P<*0.001) in individuals with the highest level of heme iron intake, compared with those with the lowest level. The pooled RR for T2DM for a daily increment of 1 mg of heme iron intake was 1.16 (1.09 to 1.23, *P<*0.001). Body iron stores, as measured by ferritin, soluble transferrin receptor (sTfR) and the sTfR:ferritin ratio, were significantly associated with the risk of T2DM. The pooled RRs for T2DM in individuals with the highest versus the lowest intake of ferritin levels was 1.70 (1.27-2.27, *P<*0.001) before adjustment for inflammatory markers and 1.63 (1.03-2.56, *P *= 0.036) after adjustment. We did not find any significant association of dietary intakes of total iron, non-heme, or supplemental iron intake with T2DM risk.

**Conclusion:**

Higher heme iron intake and increased body iron stores were significantly associated with a greater risk of T2DM. Dietary total iron, non-heme iron, or supplemental iron intakes were not significantly associated with T2DM risk.

## Background

Iron is an essential nutrient for humans, and has important metabolic functions as a cofactor for several enzymes and a major component of oxygen transporters in body. However, as a redox-active transitional metal, iron is potentially hazardous when present in excess amounts, because it catalyses several cellular reactions that result in the production of reactive oxygen species [1,2]. The pancreatic beta cells are particularly susceptible to oxidative stress because of their weak antioxidant defense [3]. Previous studies have shown that parenterally administered iron is able to induce diabetes in animals [4]. Moreover, dietary iron restriction or iron chelation has been shown to protect against developing diabetes or to attenuate the pathologic changes in diabetic models [5,6].

The association between inherited iron overload disorders and risk of type 2 diabetes mellitus (T2DM) has long been recognized in humans, based on the observation that 'secondary' T2DM is commonly (25 to 60%) complicated in patients with hereditary hemochromatosis (HH), which is characterized by progressive iron accumulation in the heart, liver, pancreas, and other organs, and by extremely high levels of circulating ferritin (typically 1000 to 10,000 ng/ml) [2]. Several recent studies indicated that moderate increases in iron stores below the levels found in patients with HH were associated with significant elevations in blood glucose and insulin levels [7,8]. Furthermore, moderately increased body iron stores at baseline were found to be significantly associated with an increased risk of future T2DM in both men [9] and women [10] in two prospective cohort studies. However, mixed findings were reported in other populations [11-19]. For instance, a more recent prospective study found no association between ferritin levels and risk of T2DM in the multivariate-adjusted models [15].

For most people, iron is obtained mainly from dietary sources and then stored in the body [2]. A cross-sectional study reported a significant association between total dietary iron intake and prevalent T2DM [18], but no such positive association was found in several other studies [20-23]. In addition, the available evidence suggests that the association between heme iron intake and risk of T2DM may be distinct from the association between non-heme iron intake and risk of T2DM [20,24].

In this study, we aimed to systematically evaluate the available evidence for associations between iron intake, body iron stores, and risk of T2DM in prospective studies, and to quantitatively summarize the data in a meta-analysis. We included only prospective studies, given the likelihood of reverse causality (possible effects of prevalent T2DM on iron intakes or measures of body iron stores) in cross-sectional or case-control studies.

## Methods

We adhered to the Meta-analysis Of Observational Studies in Epidemiology (MOOSE) guidelines [25] when undertaking this study.

### Literature search and data extraction

We identified relevant articles by a systematic search of the MEDLINE/PubMed and EMBASE databases to the end of 22 April 2012, using a combination of free text and subheadings from MeSH or EMTREE terms. The following terms were used for the MEDLINE search: ("Diabetes Mellitus/epidemiology"[Mesh] or "Hyperglycemia/epidemiology"[Mesh] or "Diabetes Mellitus, Type 2"[Mesh] or type 2 diabet*[tiab] or non-insulin dependent diabet*[tiab]) and (iron intake or iron consumption or heme iron[tiab] or iron store*[tiab] or iron status[tiab] or "Ferritins"[Mesh] or ferritin[tiab]) not (review[pt] or editorial[pt]). Similar search terms were used for the EMBASE database. In addition, the references listed in any relevant original papers and review articles were screened. No language restriction was applied for searching or study inclusion.

**Figure 1:**
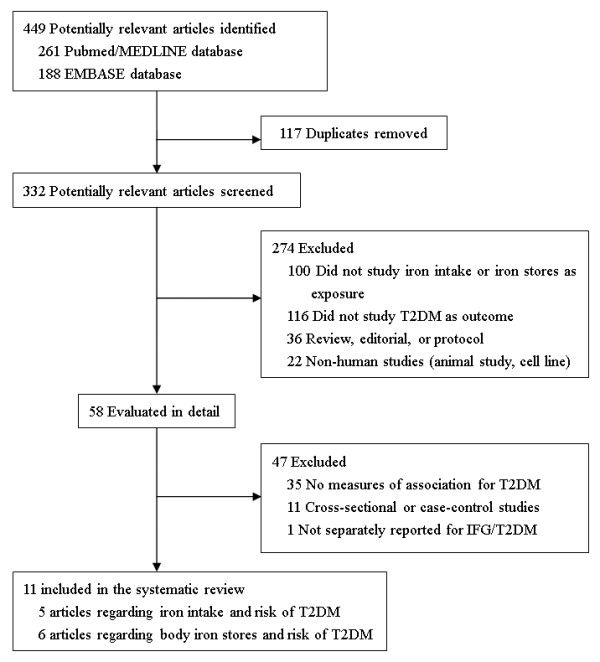
**Flow chart for study selection (through April 22, 2012)**.

Two investigators (WB and YR) independently evaluated the eligibility of all retrieved studies and extracted all relevant data using a standardized data extraction form. Any discrepancies were reconciled by consensus. A published article was included if it: 1) had a prospective cohort design, 2) evaluated the association between iron intake or body iron stores and risk of T2DM, and 3) reported the risk estimates and corresponding 95% confidence intervals (CIs) or standard errors. The process of study selection is depicted in Figure 1.

During the screening steps, several types of articles were excluded: review articles, editorials, or protocols; studies on animals or cell lines; studies that did not report iron intake or body iron stores as exposure; and studies that did not include T2DM as the outcome. In addition, studies that did not reported risk estimates or 95% CIs for the relationships between iron intake or body iron stores and risk of T2DM were excluded. One study was further excluded because data were not separately reported for T2DM [12].

For each included article, we extracted the following data: basic information (title, author, publication year, journal name), study characteristics (name of the study, study design, country, duration of follow-up), participant characteristics (sample size, number of T2DM cases, age, gender, race/ethnicity), assessment of iron intakes and body iron stores, ascertainment of T2DM, statistical methods used for the analysis, comparison, risk estimates and 95% CIs, and any covariates that were matched or adjusted for in the multivariate analysis.

If risk estimates were reported in several multivariate-adjusted models in the original studies, the one reported in the most fully-adjusted model (for example, for studies using iron intake as the exposure, we picked the model including other dietary factors as covariates) were extracted. Because circulating ferritin may be influenced by inflammation [26], we used a slightly different approach for studies that used ferritin as the indicator of body iron stores; we separately extracted the risk estimates from the most fully-adjusted models except for other biomarkers, and the models with additional adjustment for inflammation markers (such as C-reactive protein, interleukin-6, or fibrinogen). If available, we also extracted risk estimates from the models that also adjusted for other metabolic biomarkers (such as high-density lipoprotein cholesterol, triglycerides, fasting plasma glucose, fasting plasma insulin, glycated hemoglobin, homeostasis model assessment of insulin resistance, alanine aminotransferase, γ-glutamyltransferase, and adiponectin).

### Statistical analysis

Relative risk (RR), odds ratio (OR) and hazard ratio (HR) were reported as the measures of association in the included studies. Because the incidence of T2DM was sufficiently low for the rare disease assumption (<10%) to apply, the OR was assumed to approximate the RR in each case, and thus we combined the ORs with the HRs and RRs in the meta-analysis.

The RRs and corresponding 95% CIs were pooled using the random-effects model (DerSimonian-Laird method), which incorporates between-study heterogeneity in addition to sampling variation [27]. To model a dose-response trend over dietary iron intakes in association with T2DM risk, we used the method proposed by Greenland and Longnecker [28] and the publicly available Stata code written by Orsini *et al*. [29]. The dose-response results in the forest plots are presented for a daily increment of 5 mg of dietary total iron intake and a daily increment of 1 mg of heme iron intake. Because the required data for a dose-response analysis were not available in most studies of body iron stores, we did not model a dose-response trend for the association between body iron stores and T2DM risk.

Two methods were used to assess heterogeneity across studies: the χ^2^-based Cochran's Q statistic (*P<*0.10 was considered to be significant heterogeneity), and the *I*^2 ^metric (*I*^2 ^values of 25%, 50%, and 75% were considered as low, medium, and high heterogeneity, respectively) [30]. Funnel plots were used to assess small-study effects. The possibility of publication bias was assessed using the Egger regression asymmetry test [31]. For sensitivity analysis, we also used the fixed-effects model for all the above analyses. Additional sensitivity analyses were performed by omitting one study at a time and calculating a pooled estimate for the remainder of the studies to evaluate whether the results were affected markedly by a single study.

All statistical analyses were performed using Stata software (version 11.2; Stata Corp, College Station, TX, USA). All *P*-values presented are two-tailed with a significance level of 0.05, except for the Cochran's Q statistic in the heterogeneity test, in which the significance level was 0.10 [30].

## Results

### Characteristics of studies included in the meta-analysis

We identified 449 potentially relevant articles from PubMed/MEDLINE and EMBASE databases, of which 11 prospective studies [9-11,15-17,20,21,23,24,32] that met our inclusion criteria were finally included in this study. Five studies examined iron intake and risk of T2DM, while the other six assessed body iron stores and risk of T2DM.

Of the five studies that assessed iron intake and risk of T2DM [20,21,23,24,32], four were conducted in the USA and one in China. Participants were women only in three studies, men only in one study, and both women and men in one study. For assessment of iron intake, previously validated food frequency questionnaires were used in four studies, and a 3-day weighed food record method was used in the remaining study. Ascertainment of T2DM was based on self-report, plasma glucose measurements, or a combination of the two (Table 1).

**Table 1:** Characteristics of the prospective studies (n = 11) regarding the associations between iron intake or body iron stores and the risk of type 2 diabetes mellitus (T2DM)

Author, year [reference number]	Country	Study name	Sample size^a^	Age, years	Women, %	Follow-up, years	Exposure assessment	Ascertainment of T2DM
Iron intakes and T2DM (n = 5)								
Jiang *et al*, 2004 [20]	USA	HPFS	1168/38,394	40 to 75	0	12	FFQ (validated)	Symptoms plus fasting glucose, OGTT, or use of anti-diabetic medication
Lee *et al*, 2004 [24]	USA	IWHS	1921/35,698	55 to 69	100	11	FFQ (validated)	Self-report
Song *et al*, 2004 [23]	USA	WHS	1558/37,309	≥ 45	100	8.8	FFQ (validated)	Self-report
Rajpathak *et al*, 2006 [21]	USA	NHS	4599/85,031	34 to 59	100	20	FFQ (validated)	Symptoms plus fasting glucose, OGTT, or use of anti-diabetic medication
Shi *et al*, 2010 [30]	China	JIN	23/1,056	≥ 20	57.9	5	3-day weighed food records	Fasting glucose
Body iron stores and T2DM (n = 6)								
Salonen *et al*, 1998 [9]	Finland	KIHD	41/82	42 to 60	0	4	sTfR (EIA) ferritin (RIA)	Fasting glucose, OGTT, clinical diagnosis of diabetes or use of anti-diabetic treatment
Jiang *et al*, 2004 [10]	USA	NHS	698/716	56.5/56.4	100	10	sTfR (ITA); ferritin (ITA)	Symptoms plus fasting glucose, or use of anti-diabetic medication
Forouhi *et al*, 2007 [11]	UK	EPIC- Norfolk	360/758	62.4/62.1	42.0	5.1	Ferritin (FIA)	Self-report, HbA1c
Jehn *et al*, 2007 [15]	USA	ARIC	599/690	53.5/52.8	60.4	7.9	Ferritin (ITA)	Fasting or non-fasting glucose, anti-diabetic medication use, self-report
Le *et al*, 2009 [16]	USA	ACLS	220/5,292	NA	32.0	4.3-4.7	Ferritin (no details of test available)	Fasting glucose, hypoglycemic medication, or ever-diagnosed T2DM
Rajpathak *et al*, 2009 [17]	USA	DPP	280/280	50.4/50.2	63.6	2.8	Ferritin (ITA)	OGTT, fasting glucose

Abbreviations: ACLS, Aerobics Center Longitudinal Study; ARIC, Atherosclerosis Risk in Communities; DPP, Diabetes Prevention Program; EIA, enzyme immunoassay; EPIC, European Prospective Investigation into Cancer and Nutrition; FFQ, food frequency questionnaire; FIA, fluoroimmunoassay; HbA1c, glycated hemoglobin; HPFS, Health Professionals' Follow-up Study; ITA, immunoturbidimetric assay; IWHS, Iowa Women's Health Study; JIN, Jiangsu Nutrition Study; KIHD, Kuopio Ischemic Heart Disease Risk Factor Study; NHS, Nurses' Health Study; OGTT, oral glucose tolerance test; sTfR, soluble transferrin receptor; WHS, Women's Health Study.

^a ^Sample sizes were incident cases/participants for iron intake and T2DM, and cases/controls for body iron stores and T2DM.

Of the six studies that examined the association between body iron stores and risk of T2DM [9-11,15-17], four were undertaken in the USA, one in the UK, and one in Finland. One was carried out with women only, one with men only and four with both. The indicator for body iron stores was ferritin in five studies, soluble transferrin receptor (sTfR) in one study, and the ratio of sTfR to ferritin in two studies. T2DM was ascertained using plasma glucose measurements in one study, and a combination of self-reported information and plasma glucose measurements in the remaining five studies (Table 1).

### Iron intake and risk of type 2 diabetes

Associations between dietary intakes of total iron, heme iron, and non-heme iron and risk of T2DM were reported in three studies [20,21,23], five studies [20,21,23,24,32] and one study [24], respectively (Table 2). In the Iowa Women's Health Study [24], dietary total iron intake was largely reflective of non-heme iron intake (r = 0.995) and was not further analyzed, thus we derived the RR for association between dietary total iron intake and T2DM risk by pooling RRs of non-heme iron intake and heme iron intake in this study.

**Table 2:** Association between dietary and supplemental iron intakes and type 2 diabetes mellitus (T2DM) in the included studies

Source	Gender	Comparison	RRs (95% CI)	Matched or adjusted covariates
Jiang *et al*, 2004, USA [20]	Men	Dietary total iron intake: highest (median 34.2 mg/day) versus lowest (median 11.1 mg/day) quintile	1.16 (0.92 to 1.47)	Age, BMI, FH, PA, cigarette smoking, alcohol consumption, TEI, intakes of *trans *fat, cereal fiber, magnesium, whole grains, vegetables, fruit, ratio of polyunsaturated fat to saturated fat intake, glycemic load, and multivitamin use
		Dietary heme iron intake: highest (median 1.9 mg/day) versus lowest (median 0.8 mg/day) quintile	1.28 (1.02 to 1.61)	
Lee *et al*, 2004, USA [24]	Women	Dietary non-heme iron intake: highest (median 20.8 mg/day) versus lowest (median 6.5 mg/day) quintile	0.80 (0.64 to 1.01)	Age, BMI, WHR, PA, cigarette smoking status, alcohol consumption, education, marital status, residential area, hormone replacement therapy, TEI, intakes of animal fat, vegetable fat, cereal fiber, dietary magnesium, dietary non-heme iron, dietary heme iron, and supplemental iron
		Dietary heme iron intake: highest (median 2.2 mg/day) versus lowest (median 0.5 mg/day) quintile	1.28 (1.04 to 1.58)	
		Supplemental iron: highest (≥30 mg/day) versus lowest (0 mg/day) intake	1.16 (0.92 to 1.46)	
Song *et al*, 2004, USA [23]	Women	Dietary total iron intake: highest (median 33.8 mg/day) versus lowest (median 10.0 mg/day) quintile	1.13 (0.93 to 1.37)	Age, BMI, PA, FH, smoking status, alcohol consumption, TEI, dietary intakes of fiber, magnesium, and total fat, glycemic load
		Dietary heme iron intake: highest(median 1.55 mg/day) versus lowest (median 0.59 mg/day) quintile	1.46 (1.20 to 1.78)	
Rajpathak *et al*, 2006, USA [21]	Women	Dietary total iron intake: highest (median 14.0 mg/day) versus lowest (median 8.0 mg/day) quintile	1.02 (0.91 to 1.15)	Age, BMI, FH, PA, smoking status, alcohol consumption, postmenopausal hormone use, multivitamin use, TEI, intakes of cereal fiber and magnesium, caffeine, and *trans *fat (total iron, ratio of polyunsaturated to saturated fat, glycemic load, additional adjustment for intakes of whole grains, fruits and vegetables (heme iron), and additional adjustment for red meat intake (supplemental iron)
		Dietary heme iron intake: highest (median 1.9 mg/day) versus lowest (median 0.8 mg/day) quintile	1.28 (1.14 to 1.45)	
		Supplemental iron intake: highest (median 22.0 mg/day) versus lowest (median 0 mg/day) quintile	0.96 (0.84 to 1.10)	
Shi *et al*, 2010, China [30]	Both	Dietary heme iron intake: highest (median 4.4 mg/day) versus lowest (median 0.1 mg/day) quartile	9.84 (1.41 to 68.75)	Age, gender, BMI, central obesity, hypertension, FH, PA, sedentary behavior, cigarette smoking, alcohol consumption, TEI, intakes of fat, fiber and magnesium, education, income, and job

Abbreviations: BMI, body mass index; FH, family history of diabetes; PA, physical activity; RR, relative risk; TEI, total energy intake; WHR, waist:hip ratio.

**Figure 2:**
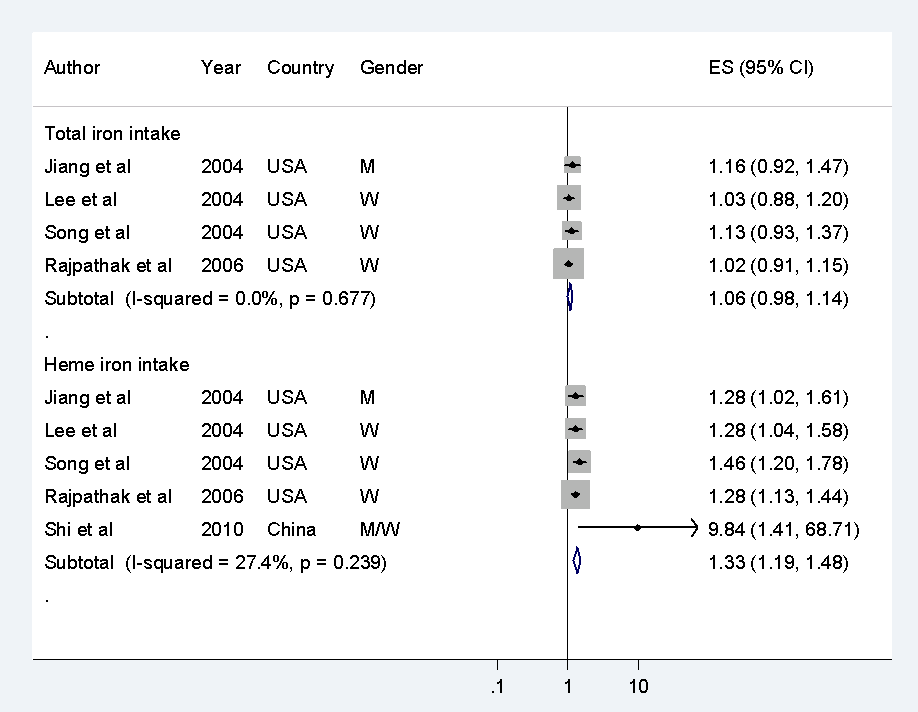
**Associations between dietary total iron, heme iron intake and risk of type 2 diabetes mellitus (T2DM) in the included studies, comparing the highest category with the lowest**. The risk estimate of dietary total iron intake for T2DM risk in the Iowa Women's Health Study (Lee *et al *[24]) was not directly reported, and thus this was pooled from the results of dietary non-heme iron intake and heme iron intake in this study. M, men; W, women.

A meta-analysis of five studies showed that higher intake of heme iron was associated with a significantly greater risk of T2DM. The pooled RR (95% CI) in individuals with the highest level of heme iron intake compared with those with the lowest levels was 1.33 (1.19 to 1.48, *P<*0.001) for T2DM (Figure 2). However, no significant association was seen between dietary total iron intake and risk of T2DM; the pooled RRs and 95% CIs comparing the highest with the lowest levels of dietary total iron intake was 1.06 (0.98 to 1.14, *P *= 0.18) (Figure 2). There was no evidence for significant heterogeneity (heme iron intake: *P *= 0.24 (Q statistic), *I*^2 ^= 27%; dietary total iron intake: *P *= 0.68 (Q statistic), *I*^2 ^= 0%) or publication bias (heme iron intake, *P *= 0.08 (Egger test); dietary total iron intake, *P *= 0.06 (Egger test)) across the included studies (see Additional file 1, Figure S1).

**Figure 3:**
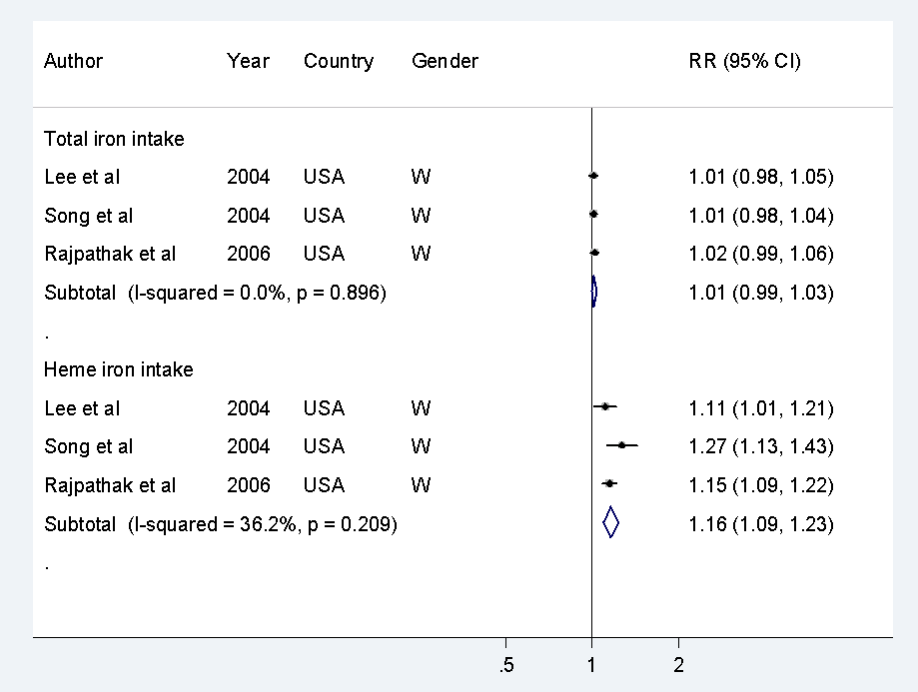
**Dose-response analyses of dietary intakes of total iron and heme iron in relation to risk of type 2 diabetes mellitus (T2DM) in the included studies**. The risk estimate of dietary total iron intake for T2DM risk in the Iowa Women's Health Study (Lee *et al *[24])) was not directly reported, and thus this was pooled from the results of dietary non-heme iron intake and heme iron intake in this study. W, women.

In the dose-response analyses (Figure 3), the pooled RRs of T2DM were 1.16 (1.09 to 1.23, *P<*0.001) for a 1 mg/day increment of heme iron intake, with no evidence of heterogeneity (I^2^ = 36%, *P* = 0.21), and 1.01 (0.99 to 1.03) for each 5 mg/day increment of dietary total iron intake, with no evidence of heterogeneity (I^2^ = 0%, *P* = 0.90).

Sensitivity analyses using a fixed-effects model yielded similar results, and omitting one study at a time did not substantially alter the pooled results. In addition, two studies reported associations between supplemental iron intake and risk of T2DM [21,24], and one study reported an association between the sum of dietary iron intake and supplemental iron intake and risk of T2DM [21]. However, neither of these studies found a significant association between supplemental iron or the summed iron intake and the risk of T2DM.

### Body iron stores and risk of type 2 diabetes

A significant association between body iron stores and risk of T2DM was seen in three [10,11,16] of the five studies that used ferritin as the indicator, and in all studies that used the sTfR:ferritin atio [9,10] or the sTfR [17] alone as the indicator (Table 3).

**Table 3:** Association between body iron stores and type 2 diabetes mellitus (T2DM) in the included studies

Source	Gender	Comparison	Models	RR (95% CI)	Matched or adjusted covariates
Ferritin as indicators of body iron stores (n = 5)					
Jiang *et al*, 2004, USA [10]	Women	Highest (≥107.2 ng/ml) versus lowest (<21.1 ng/ml) quintile	Model 1^a^	2.68 (1.75 to 4.11)	Age, ethnicity, fasting status, BMI, FH, PA, smoking, alcohol consumption, menopausal status, glycemic load, intake of total energy, cereal fiber, magnesium, and *trans *fat, and ratio of polyunsaturated fat to saturated fat
			Model 2^b^	2.61 (1.68 to 4.07)	Additional adjustment for CRP
Forouhi *et al*, 2007, UK^c ^[11]	Men	Highest (≥135.7 ng/ml) versus lowest (<34.4 ng/ml) quintile	Model 1^a^	1.97 (1.12 to 3.45)	Age, sex, BMI, FH, PA, smoking, dietary factors (TEI, alcohol consumption, intake of dietary iron, magnesium, and red meat and processed meat, plasma vitamin C)
			Model 2^b^	1.78 (0.99 to 3.19)	Additional adjustment for CRP, fibrinogen, and IL-6
			Model 3	1.13 (0.58, 2.19)	Additional adjustment for ALT, GGT, and adiponectin
	Women	Highest (≥71.7 ng/ml) versus lowest (<17.8 ng/ml) quintile	Model 1^a^	2.55 (1.22 to 5.34)	Age, sex, BMI, FH, PA, smoking, dietary factors (TEI, alcohol consumption, intake of dietary iron, magnesium, red meat and processed meat and plasma vitamin C)
			Model 2^b^	2.11 (0.98 to 4.56)	Additional adjustment for CRP, fibrinogen, and IL-6
			Model 3	1.08 (0.44, 2.62)	Additional adjustment for ALT, GGT, and adiponectin
Jehn *et al*, 2007, USA [15]	Both	Highest (≥235.4 ng/ml; median, 354.5 ng/ml) versus lowest (<40.0 ng/ml; median, 20.0 ng/ml)quintile	Model 1^a^	1.51 (0.98 to 2.31)	Age, study center, ethnicity, smoking, alcohol consumption, and BMI
			Model 2	0.81 (0.49 to 1.34)	Additional adjustment for metabolic syndrome components (HDL-C, WC, hypertension, FPG, and TG)
			Model 3^b^	0.79 (0.48 to 1.32)	Additional adjustment for FPI and inflammation score
Le *et al*, 2009, USA [16]	Men	Highest (>188 ng/ml) versus lowest (<80 ng/ml) quartile	Model 1^a^	1.79 (1.13 to 2.82)	Age, ethnicity, and BMI
	Women	Highest (premenopausal, >60 ng/ml, postmenopausal, >90 ng/ml) versus lowest (premenopausal,*<*21 ng/ml, postmenopausal,*<*37 ng/ml) quartile	Model 1^a^	0.87 (0.37 to 2.03)	Age, ethnicity, and BMI
Rajpathak *et al*, 2009, USA [17]	Both	Highest (median, 203.7 ng/ml) versus lowest (median, 20.1 ng/ ml) quartile	Model 1^a^	1.02 (0.60 to 1.74)	Age, sex, ethnicity, and BMI
			Model 2	1.65 (0.90 to 3.02)	Additional adjustment for FH, PA, HbA1c, and sTfR
			Model 3^b^	1.53 (0.83 to 2.82)	Additional adjustment for CRP
			Model 4	1.61 (0.85 to 3.02)	Additional adjustment for HOMA-IR
Ratio of sTfR to ferritin as indicators of body iron stores (n = 2)					
Salonen *et al*, 1998, Finland [9]	Men	Highest (< 9.4) versus quartile (no data available)	Model 1	2.40 (1.03 to 5.50)	Age, time of examination, place of residence, cigarette smoking, exercise, maximal oxygen uptake, socioeconomic status, height, weight, hip and waist circumferences, glucose, insulin, vitamin E, and serum SFA to (PUFA + MUFA) ratio
Jiang *et al*, 2004, USA [10]	Women	Highest (<26.7) versus lowest (≥149.4) quintile	Model 1	2.44 (1.61 to 3.71)	Age, ethnicity, fasting status, BMI, FH, PA, smoking, alcohol consumption, menopausal status, glycemic load, intake of total energy, cereal fiber, magnesium, and *trans *fat, ratio of polyunsaturated fat to saturated fat
			Model 2	2.40 (1.55 to 3.71)	Additional adjustment for CRP
sTfR as indicators of body iron stores (n = 1)					
Rajpathak *et al*, 2009, USA [17]	Both	Highest (median, 4.4 mg/l) versus lowest (median, 2.3 mg/l) quartile	Model 1	1.55 (0.93 to 2.57)	Age, sex, ethnicity, and BMI
			Model 2	2.26 (1.27 to 4.01)	Additional adjustment for FH, PA, HbA1c, and sTfR
			Model 3	2.39 (1.34 to 4.28)	Additional adjustment for CRP
			Model 4	2.23 (1.22 to 4.06)	Additional adjustment for HOMA-IR

Abbreviations: ALT, alanine aminotransferase; BMI, body mass index; CRP, C-reactive protein; FH, family history; FPG, fasting plasma glucose; FPI, fasting plasma insulin; GGT, γ-glutamyltransferase; HbA1c, glycated hemoglobin; HDL-C, high-density lipoprotein cholesterol; HOMA-IR, homeostasis model assessment for insulin resistance; IL-6, interleukin-6; MUFA, monounsaturated fatty acids; PA, physical activity; PUFA, polyunsaturated fatty acids; RR, relative risk; SFA, saturated fatty acids; sTfR, soluble transferrin receptor; TEI, total energy intake; TG, triglycerides; WC, waist circumference.

^a ^Estimates used in the meta-analysis of ferritin and T2DM risk in multivariate-adjusted models.

^b ^Estimates used in the meta-analysis of ferritin and T2DM risk in multivariate-adjusted models including inflammatory markers.

^c ^From an *ad hoc *analysis by Forouhi *et al*. [11], which used quintiles of ferritin levels as exposure and separately reported for men and women.

**Figure 4:**
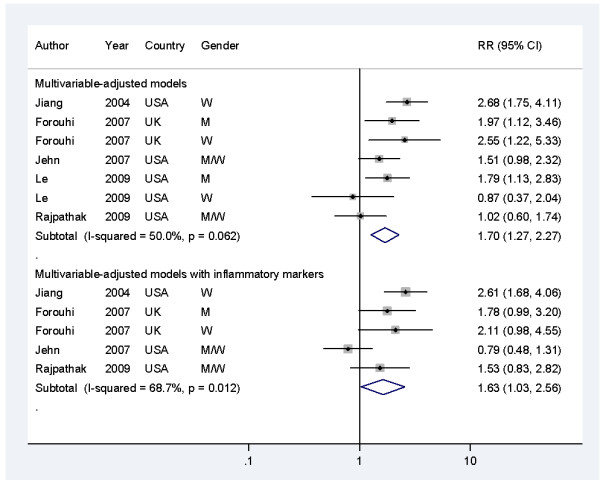
**Associations between circulating ferritin levels and risk of type 2 diabetes mellitus (T2DM) in the included studies**. M indicates men and W for women. The data in the EPIC-Norfolk study [11] were the results of an *ad hoc *analysis by the original authors, which used quintiles of ferritin levels as exposure and separately reported for men and women. M, men; W, women.

For the meta-analysis of studies using ferritin as the indicator, the pooled RR (95% CI) for T2DM was 1.70 (1.27 o 2.27, *P<*0.001) for individuals with the highest ferritin levels, compared with those with the lowest (Figure 4). In the heterogeneity test, there was greater variation (moderate to high) in the study results than would be expected as a result of chance (*P *= 0.06 (Q statistic), *I*^2 ^= 50%), which is not surprising given the substantial differences in study populations and methods. No evidence of publication bias was found (see Additional file 1, Figure S2A, *P *= 0.49 (Egger test)). Sensitivity analyses using fixed-effect model or omitting one study at a time did not substantially alter the pooled results.

Because measurement of circulating ferritin levels may be confounded by inflammation [26], we performed additional meta-analysis of the studies that reported RRs in multivariate-adjusted models with additional adjustment for inflammatory markers. The pooled RR (95% CI) for T2DM was 1.63 (1.03 to 2.56, *P *= 0.04) comparing individuals with the extreme categories (Figure 3). The heterogeneity across studies was high (*P *= 0.01 (Q statistic), *I*^2 ^= 69%), which might be explained by the inclusion of other metabolic markers that could be biological intermediates in the multivariate models of several studies [15,17]. A stratification analysis showed that the pooled RRs (95% CI) for T2DM were 2.24 (1.63 to 3.09) and 1.07 (0.56 to 2.05) for studies without and with other metabolic markers in the models, respectively. No evidence of publication bias was seen (*P *= 0.93 (Egger test) (see Additional file 1, Figure S2B)). Sensitivity analyses using a fixed-effects model yielded similar results, and omitting one study at a time did not substantially alter the magnitude of association, except for the omission of the study by Jehn *et al*. [15] (the pooled RR (95% CI) of the remaining studies was 2.06 (1.55 to 2.74)).

## Discussion

### Summary of main findings

In this study, we found that heme iron intake and body iron stores were positively associated with an increased risk of T2DM, after adjustment for known potential confounders. Although dietary total iron intake has been associated with prevalent T2DM in a cross-sectional study [18], our meta-analysis of prospective studies found no significant association of dietary intakes of total iron, non-heme, and supplemental iron intake with the risk of T2DM. The positive association we found between heme iron and T2DM was consistent with previous results from prospective studies evaluating the relationships between red meat (the major source of heme iron) and the risk of T2DM [33,34]. Because heme iron intake was found to be associated with higher body iron stores in previous studies [35,36], our results suggest that high levels of body iron stores may mediate the association of increased risk of T2DM with chronically high heme iron intake.

### Interpretation

The positive association between excess iron and risk of T2DM is biologically plausible, although the underlying mechanisms still remain to be fully determined. First of all, iron is a powerful pro-oxidant and catalyst that promotes the formation of hydroxyl radicals, which may attack pancreatic beta cells through increasing oxidative stress and thus result in impaired insulin synthesis and excretion [37]. Antioxidant enzymes, such as superoxide dismutase, catalase, and glutathione peroxidase, are expressed less in pancreatic islets than in other tissues, thus pancreatic islets are particularly susceptible to oxidative damage [3]. In a mouse model of hemochromatosis, excess iron resulted in beta-cell oxidant stress and decreased insulin secretory capacity secondary to beta-cell apoptosis and desensitization of glucose-induced insulin secretion [38]. Another study showed that even at 'normal' levels, iron exerted detrimental effects on pancreatic beta-cell function, and that these effects were reversible with dietary restriction or iron-chelation therapy [5]. Second, excess iron may diminish glucose utilization in muscle tissue and lead to a shift from glucose to fatty acid oxidation, which may result in increased insulin resistance. In a recent study using a mouse model of hemochromatosis, although glucose uptake was increased in skeletal muscle, glucose oxidation was decreased and the ratio of fatty acid to glucose oxidation was increased, as a result of decreased pyruvate dehydrogenase (PDH) enzyme activity and increased expression of PDH kinase 4 [39]. Third, increased substrate recycling to the liver may contribute to the higher hepatic glucose production [39].

Heme iron exists only in meat and meat products, where it occurs in the form of hemoglobin and myoglobin, whereas non-heme iron is present in both plant and animal foods [40]. The regulation of the intestinal iron absorption is crucial because there is no physiologic mechanism for excretion of iron [1]. Although heme iron is not the predominant form of dietary iron, it more readily absorbed than non-heme iron [2]. In addition, absorption of heme iron does not change with increasing dose, is less substantially affected by iron status than non-heme iron, and is not greatly influenced by other components in diet [35,40]. Therefore, heme iron contributes about 50% of the total bioavailable iron in the typical western diet [20]. In an elderly population of the Framingham Heart Study, non-heme iron was the major source (mean 93%) of iron, yet heme iron intake was significantly and positively associated with serum ferritin concentration, whereas non-heme iron intake did not significantly influence serum ferritin concentration [35]. Similar positive association between heme iron intake and serum ferritin concentration was reported from the Health Professionals' Follow-up Study [41]. Therefore, the distinct effects of heme iron and non-heme iron intake on risk of T2DM that we found in the present study may be, at least partly, explained by the differences in bioavailability of the different iron types and their effects on body iron stores.

Circulating ferritin, a major iron storage protein in body, has been widely used as an index of body iron stores [26]. However, the specificity of high circulating ferritin levels as a marker of increased body iron stores is somewhat limited because ferritin is an acute-phase reactant [2], and circulating ferritin is increased in the presence of inflammation and other disorders [26,42]. In addition, circulating ferritin is also increased with alcohol consumption and body mass index (BMI), and differs with gender [35,36]. In our meta-analysis, even though we adjusted for alcohol consumption, BMI, gender, inflammation and other factors, the association between ferritin and risk of T2DM remained significant. In addition, the sTfR:ferritin ratio has been found to distinguish between subjects with similarly high ferritin levels, and sTfR is believed to be free of influence by acute or chronic inflammation, therefore it has been suggested that the sTfR:ferritin ratio is a better marker than ferritin alone to measure a wide range of iron levels [43]. Thus far, two prospective studies using the sTfR:ferritin ratio as a marker of body iron stores found similar significant associations [9,10]. Use of phlebotomy or iron-chelation therapy to reduce ferritin levels was associated with improved glucose tolerance in patients with HH [44,45], healthy blood donors [46], patients with metabolic syndrome [47], and patients with T2DM [48]. These studies support our findings of a significant association between increased body iron stores and T2DM risk.

### Strengths and limitations

The strengths of our study include the use of prospective studies with long-term follow-up, large sample size, and extensive adjustment for potential confounders, which together reduce the possibility of selection bias and reverse causation. However, several limitations need to be addressed. First, genetic polymorphisms (for example, H63D and C282Y variants in the *HFE *gene, which contributes to the development of HH) may affect the efficiency of iron absorption and body iron stores [40]. A potential interaction between *HFE *genotypes and heme iron intake in relation to the risk of T2DM has been reported in a previous study [49]. However, the allele frequencies of *HFE *variants are very low in the general population [49] and thus there is a low likelihood that they would have substantially affected the observed associations in the present study. In addition, our previous studies found that microsatellite polymorphism in the heme oxygenase-1 gene (*HO-1*), which encodes the rate-limiting enzyme in heme iron catabolism, was associated with a higher risk of T2DM [50,51]. The possible interaction between heme iron intake and *HO-1 *gene polymorphisms and their combined effects in the pathogenesis of T2DM remain unknown. Second, a high correlation between intake of heme iron and red meat (the latter as a major contributor to heme iron intake that has been associated with higher risk of T2DM [34]) was found in the included study [23]. Because of a high degree of statistical collinearity, we were unable to reliably separate the independent effect of heme iron *per se *from other components of red meat. However, the potential link of T2DM risk to heme iron cannot be excluded because it is biologically plausible. Third, comparing the highest category of exposure with the lowest for each study cannot fully quantify the association. The dose-response trend of body iron stores in relation to T2DM risk should be evaluated in future when more studies are available. Fourth, the number of studies was not sufficient to allow us to conduct a formal meta-regression analysis for identifying potential sources of heterogeneity, and this also limited an adequate assessment of small-study effects. Fifth, most of the included studies were conducted in western populations, which limits the direct generalization of the findings. Although Asia has the largest number of patients with diabetes in the world [52,53], and several studies in East Asian populations have suggested that T2DM were more prevalent among people with higher heme iron intakes [22,32] and body iron stores [14,19,22], more studies, in particular prospective cohort studies, are still required in this population of high diabetes prevalence. Finally, although many potential confounders have been adjusted for in the included studies, we cannot completely exclude the possibilities of residual confounding from unmeasured or incompletely measured factors.

## Conclusions

In conclusion, higher heme iron intake and higher body iron stores were significantly associated with a greater risk of T2DM in this meta-analysis of prospective studies. It may therefore be necessary to reconsider the dietary reference values for iron [40], in particular in countries where most of the population ingest sufficient iron and/or are at risk of iron overload. Dietary total iron, non-heme iron, or supplemental iron intakes were not significantly associated with T2DM risk. Moderately increased ferritin levels may be useful for clinical and public health identification of high-risk groups for T2DM; however, further studies are warranted.

## Abbreviations

ALT: alanine aminotransferase; BMI: body mass index; CI: confidential interval; CRP: C-reactive protein; FFQ: food frequency questionnaire; FPG: fasting plasma glucose; FPI: fasting plasma insulin; GGT: γ-glutamyltransferase; HbA1c: glycated hemoglobin; HDL-C: high-density lipoprotein cholesterol; HH: hereditary hemochromatosis; HO-1: heme oxygenase-1; HOMA-IR: homeostasis model assessment of insulin resistance; HR: hazard ratio; OGTT: oral glucose tolerance test; OR: odds ratio; PDH: pyruvate dehydrogenase; RR: relative risk; sTfR: soluble transferrin receptor; T2DM: type 2 diabetes mellitus; TC: total cholesterol; TG: triglycerides.

## Competing interests

The authors declare that they have no competing interests.

## Authors' contributions

WB and LL conceived the idea and designed the study. WB, YR and SR collected data, conducted data analysis and interpreted the results. WB wrote the manuscript. YR and SR and LL revised the manuscript critically for important intellectual content, and approved the final version of the manuscript. All authors read and approved the final manuscript.

## Pre-publication history

The pre-publication history for this paper can be accessed here:

http://www.biomedcentral.com/1741-7015/10/119/prepub

## Supplementary Material

Additional file 1 **Funnel plots**. Associations between **(S1) **intakes of total iron, heme iron, and risk of type 2 diabetes mellitus (T2DM) and **(S2) **associations between ferritin levels and risk of T2DM before and after adjusting for inflammatory markers. Click here for file
