# Supplementary material for: Dietary iron intake, body iron stores, and the risk of type 2 diabetes: a systematic review and meta-analysis
Source: BMC Med. 2012 Oct 10;10:119. doi: 10.1186/1741-7015-10-119 (PMC3520769; doi:10.1186/1741-7015-10-119)
Supplement: Additional file 1 — Funnel plots. Associations between (S1) intakes of total iron, heme iron, and risk of type 2 diabetes mellitus (T2DM) and (S2) associations between ferritin levels and risk of T2DM before and after adjusting for inflammatory markers. [file 1741-7015-10-119-S1.DOCX]

**Additional file 1**

**Figure Legends**

**Figure S1.** Funnel plots for the associations between intakes of total iron (A), heme iron (B) and risk of T2DM in the included studies.

**Figure S2.** Funnel plots for the association between ferritin levels and risk of T2DM in the included studies, before (A) and after (B) adjusting inflammatory markers.

(A)

(B)

**Figure S1.** Funnel plots for the associations between intakes of total iron (A), heme iron (B) and risk of T2DM in the included studies.

(A)

(B)

**Figure S2.** Funnel plots for the association between ferritin levels and risk of T2DM in the included studies, before (A) and after (B) adjusting inflammatory markers.
